# Analysis of Human Leukocyte Antigen DR Alleles, Immune-Related Adverse Events, and Survival Associated With Immune Checkpoint Inhibitor Use Among Patients With Advanced Malignant Melanoma

**DOI:** 10.1001/jamanetworkopen.2022.46400

**Published:** 2022-12-13

**Authors:** Halis Kaan Akturk, Kasey L. Couts, Erin E. Baschal, Kagan E. Karakus, Robert J. Van Gulick, Jacqueline A. Turner, Laura Pyle, William A. Robinson, Aaron W. Michels

**Affiliations:** 1 Barbara Davis Center for Diabetes, University of Colorado School of Medicine, Aurora; 2 Department of Pediatrics, University of Colorado School of Medicine, Aurora; 3 Department of Medicine, University of Colorado School of Medicine, Aurora; 4 University of Colorado Cancer Center, University of Colorado School of Medicine, Aurora; 5 Department of Biostatistics and Informatics, Colorado School of Public Health, Aurora; 6 Department of Immunology, University of Colorado School of Medicine, Aurora

## Abstract

**Importance:**

Treatment with immune checkpoint inhibitors (ICIs) has increased survival in patients with advanced malignant melanoma but can be associated with a wide range of immune-related adverse events (irAEs). The role of human leukocyte antigen (HLA)–DR alleles in conferring irAE risk has not been well studied.

**Objective:**

To evaluate the association between irAEs and treatment response, survival, and the presence of HLA-DR alleles after ICI therapy in advanced melanoma.

**Design, Setting, and Participants:**

This case-control study used the patient registry and biobanked samples from the tertiary referral University of Colorado Cancer Center. Specimens and clinical data were collected between January 1, 2010, and December 31, 2021. Patients with advanced (stage III unresectable and stage IV) melanoma who received ICI therapy (n = 132) were included in the analysis.

**Exposures:**

Immune checkpoint inhibitors (anti–cytotoxic T-lymphocyte antigen 4, anti–programmed cell death protein 1 or its ligand, or the combination) for the treatment of advanced melanoma.

**Main Outcomes and Measures:**

The association between irAEs and response to therapy, survival, and HLA-DR alleles.

**Results:**

Among the cohort of 132 patients with advanced melanoma (mean [SD] age, 63.4 [7.2] years; 85 men [64%] and 47 women [36%]) treated with ICIs, 73 patients had at least 1 irAE and 59 did not have an irAE. Compared with patients without an irAE, patients with an irAE had higher treatment response rates (50 of 72 [69%] vs 28 of 57 [49%]; *P* = .02) and increased survival (median, 4.8 [IQR, 0.2-9.6] vs 3.2 [IQR, 0.1-9.2] years; *P* = .02). Specific HLA-DR alleles were associated with the type of irAE that developed: 7 of 10 patients (70%) who developed type 1 diabetes had DR4; 6 of 12 (50%) who developed hypothyroidism had DR8; 5 of 8 (63%) who developed hypophysitis had DR15; 3 of 5 (60%) who developed pneumonitis had DR1; and 8 of 15 (53%) who developed hepatitis had DR4.

**Conclusions and Relevance:**

These findings suggest that IrAEs are associated with treatment response rates and increased survival after ICI therapy for advanced melanoma. Because distinct HLA-DR alleles are associated with given adverse events, HLA genotyping before ICI therapy may aid in identifying risk for specific irAEs that could develop with such treatment.

## Key Points

**Question:**

What is the association between immune-related adverse events (irAEs) and survival after immune checkpoint inhibitor (ICI) therapy initiation and human leukocyte antigen genes in patients with advanced malignant melanoma?

**Findings:**

In this case-control study of 132 adults with advanced malignant melanoma treated with ICIs, patients with irAEs had improved treatment response rates and better survival. Distinct HLA-DR alleles were associated with specific irAEs.

**Meaning:**

These findings suggest that the occurrence of irAEs after ICI therapy in patients with advanced melanoma is associated with improved treatment response, survival, and certain HLA-DR alleles.

## Teaser

This case-control study examines the association between development of immune-related adverse events (irAEs) and response to therapy, survival, and human leukocyte antigen (HLA) class II genes in patients with advanced melanoma treated with immune checkpoint inhibitors (ICIs).

## Introduction

Immune checkpoint inhibitors (ICIs) have significantly transformed the treatment of advanced-stage melanoma and other cancers; however, a wide range of immune-related adverse events (irAEs) can result from treatment, especially those targeting self-tissues.^1,2^ Notable irAEs after ICI therapy include colitis, hypothyroidism, hepatitis, pneumonitis, vitiligo, type 1 diabetes (the immune-mediated form of diabetes), and hypophysitis (pituitary gland inflammation).^3^ Previous studies have shown associations between the specific immune checkpoint receptor being targeted and the irAEs that develop. Cytotoxic T-lymphocyte antigen 4 (CTLA-4) inhibitors increase the risk of hypophysitis, and programmed cell death protein 1 (PD-1) and its ligand (PD-L1) inhibitors are associated with autoimmune thyroid disease and type 1 diabetes.^4,5,6,7^ Interestingly, human leukocyte antigen (HLA) class II genes confer significant risk for the development of corresponding autoimmune disorders outside the use of ICI therapy.^8^ However, little is known regarding the HLA antigen basis for development of specific irAEs and survival after these unwanted effects. In this study, we examined the association between development of irAEs and the response to therapy, survival, and HLA class II genes in patients with advanced melanoma treated with ICIs.

## Methods

### Study Population

Patients 18 years or older with pathologically confirmed advanced melanoma (stage III unresectable or stage IV disease) who received 1 or more doses of anti–PD-1 or anti–CTLA-4 monotherapy, or any combination, were included in this study using the Skin Cancer Biorepository patient registry and biobanked samples at the University of Colorado Cancer Center. Written informed consent was obtained from each participant, and the Colorado Multiple Institutional Review Board approved the study. This study followed the Strengthening the Reporting of Observational Studies in Epidemiology (STROBE) reporting guideline.

### Data Collection

One hundred thirty-two consecutive adult patients with advanced melanoma treated with ICI therapy between January 1, 2010, and December 31, 2021, were assessed for irAEs and underwent HLA-DR genotyping. The irAEs were diagnosed by the treating oncology team based on pathologic evidence and/or supporting laboratory evidence with multidisciplinary adjudication. Treatment response rates are based on the iRECST (immune Response Evaluation Criteria in Solid Tumors) criteria, which applies a standard approach to the measurement of solid tumors and defines an objective change in tumor size.^9^ Stored DNA samples from peripheral blood samples were used to perform high-resolution 4-digit genotyping of HLA-DRB1 alleles using oligonucleotide probes.^10^

### Statistical Analysis

Statistical analyses were performed using GraphPad Prism software, version 9.2.0 (GraphPad), with 2-sided *P* < .05 indicating statistical significance. Kaplan-Meier curves were used to evaluate survival after ICI therapy initiation, with *P* values calculated using a log-rank test. Fisher exact tests were used to compare proportions of patients with and without a treatment response and patients who had an irAE with a specific HLA-DR allele with those who had no irAE and the same HLA-DR allele.

## Results

In our case-control cohort of 132 patients with advanced melanoma (mean [SD] age, 63.4 [7.2] years; 85 men [64%] and 47 women [36%]) treated with ICIs, 73 had an irAE, and 59 had no irAE. Both groups were comparable in terms of age, sex, and use of ICI agents, including combination therapy (Table). The most frequent irAEs among all patients were hepatitis (16 [12%]), hypothyroidism (14 [11%]), type 1 diabetes (12 [9%]), vitiligo (8 [6%]), hypophysitis (8 [6%]), and pneumonitis (5 [4%]).

**Table.** Characteristics of Patients With Advanced Melanoma With and Without irAEs After Initiation of ICI Therapy^a^

Characteristic	irAE present		Type of irAE					
	No (n = 59)	Yes (n = 73)	Hepatitis (n = 16)	Hypothyroidism (n = 14)	Type 1 diabetes (n = 12)	Vitiligo (n = 8)	Hypophysitis (n = 8)	Pneumonitis (n = 5)
Age, y								
Mean (SD)	64.1 (11.1)	58.7 (5.6)	62.1 (3.5)	61.4 (7.1)	58.3 (3.8)	59.9 (7.3)	62.1 (3.5)	63.2 (2.5)
Median (range)	61 (22-90)	59 (19-66)	60 (50-68)	60 (47-78)	56 (19-79)	56 (51-73)	59 (55-69)	60 (59-68)
Sex								
Men	39 (66)	46 (63)	11 (69)	9 (64)	8 (67)	6 (75)	6 (75)	5 (100)
Women	20 (34)	27 (37)	5 (31)	5 (36)	4 (33)	2 (25)	2 (25)	0
ICI therapy								
Anti–PD-1/PD-L1	17 (29)	18 (25)	2 (12)	5 (36)	4 (33)	2 (25)	0	0
Anti–CTLA-4	6 (10)	2 (3)	0	0	0	0	0	0
Combination	36 (61)	53 (73)	14 (88)	9 (64)	8 (67)	6 (75)	8 (100)	5 (100)
Overall survival, y								
Mean (SD)	3.6 (2.6)	4.6 (2.3)	4.6 (1.9)	3.9 (1.6)	3.9 (2)	4.1 (1.8)	4 (2.4)	4.4 (1.6)
Median (range)	3.2 (0.1-9.2)	4.8 (0.2-9.6)	4.4 (1.3-7.7)	3.8 (1.5-7.9)	3.8 (1.4-7.7)	4.3 (1.3-6.9)	4.7 (0.7-6.7)	4.4 (2.5-6.8)
Treatment response^b^	28 (49)	50 (69)	12 (80)	11 (85)	11 (92)	4 (50)	3 (38)	3 (60)
HLA-DR allele present^c^								
DR1	12 (24)	10 (15)	1 (7)	2 (14)	2 (20)	1 (13)	0	3 (60)
DR3	13 (26)	12 (16)	2 (13)	1 (8)	1 (10)	1 (13)	1 (13)	0
DR4	15 (30)	16 (24)	8 (53)	5 (42)	7 (70)	3 (38)	2 (25)	0
DR7	8 (16)	16 (24)	2 (13)	1 (8)	1 (10)	1 (13)	3 (38)	0
DR8	4 (8)	10 (15)	2 (13)	6 (50)	2 (20)	2 (25)	0	2 (40)
DR9	1 (2)	3 (4)	0	1 (8)	0	0	0	0
DR10	0	2 (3)	1 (7)	0	0	0	0	0
DR11	8 (16)	10 (15)	3 (20)	1 (8)	3 (30)	0	1 (13)	1 (20)
DR12	1 (2)	2 (3)	0	1 (8)	0	0	0	0
DR13	15 (30)	15 (22)	2 (13)	3 (25)	2 (20)	2 (25)	2 (25)	2 (40)
DR14	1 (2)	4 (6)	3 (20)	0	1 (10)	2 (25)	0	0
DR15	13 (26)	17 (25)	4 (27)	2 (17)	0	3 (38)	5 (63)	2 (40)
DR16	1 (2)	2 (3)	1 (7)	1 (8)	0	1 (13)	0	0

Abbreviations: CTLA-4, cytotoxic T-lymphocyte antigen 4; HLA, human leukocyte antigen; ICI, immune checkpoint inhibitor; irAE, immune-related adverse event; PD-1/PD-L1, programmed cell death 1 and its ligand.

^a^ The most frequent irAEs are included in the Table; several patients have more than 1 irAE. Unless indicated otherwise, data are expressed as No. (%) of patients. Owing to rounding, some subsets may not total 100%.

^b^ Treatment response was determined using Immune Response Evaluation Criteria in Solid Tumors in 57 patients with no irAE and 72 with any irAE, including 15 with hepatitis, 13 with hypothyroidism, 12 with type 1 diabetes, 8 with vitiligo, 8 with hyophysitis, and 5 with pneumonitis.

^c^ HLA-DR genotyping was performed in 50 patients with no irAE and 67 with any irAE, including 15 with hepatitis, 12 with hypothyroidism, 10 with type 1 diabetes, 8 with vitiligo, 8 with hyophysitis, and 5 with pneumonitis.

We first evaluated the association between ICI response rates and irAEs. The treatment response rate was significantly higher in patients with at least 1 irAE compared with patients without irAEs (50 of 72 [69%] vs 28 of 57 [49%]; *P* = .02). Additionally, the development of type 1 diabetes (11 of 12 [92%]), hepatitis (12 of 15 [80%]), or hypothyroidism (11 of 13 [85%]) was associated with ICI response rates of at least 80% (Table).

**Figure 1.**
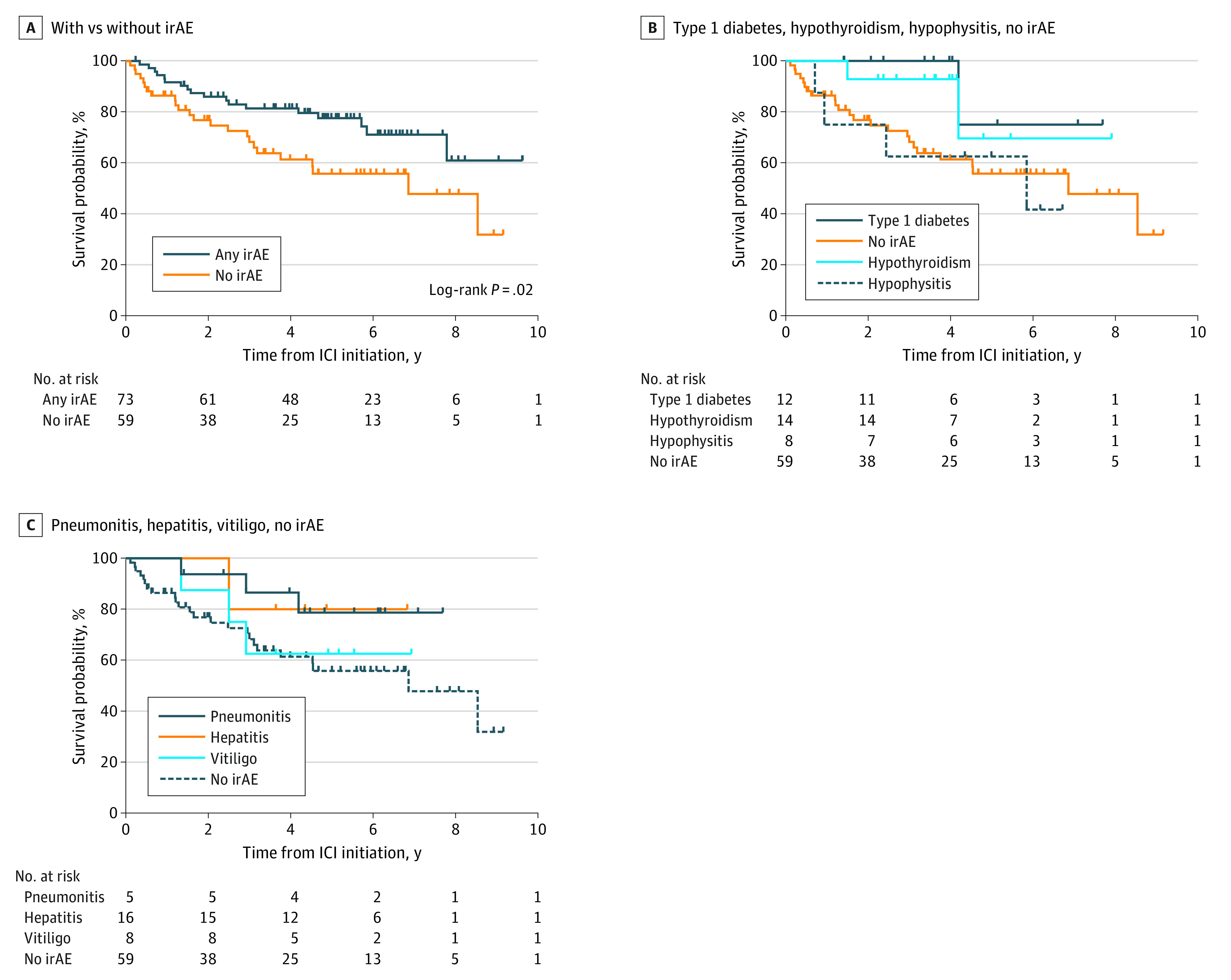
Survival in Patients With Advanced Melanoma by the Presence of Immune-Related Adverse Events (irAEs) Kaplan-Meier plots of survival probability after initiation of immune checkpoint inhibitor (ICI) treatment in patients with or without an irAE (A) and by specific irAE (B and C).

Next, we examined the association between survival from the start of ICI therapy and the occurrence of irAEs. Patients with an irAE had a significantly longer survival compared with those without an irAE (median, 4.8 [IQR, 0.2-9.6] vs 3.2 [IQR, 0.1-9.2] years; *P* = .02) (Figure 1A). There was also a trend toward patients with specific irAEs having improved survival compared with those without an irAE (eg, median survival for patients with hepatitis, 4.4 [IQR, 1.3-7.7] years; median survival for patients with hypothyroidism, 3.8 [IQR, 1.5-7.9] years) (Figure 1B and C).

**Figure 2.**
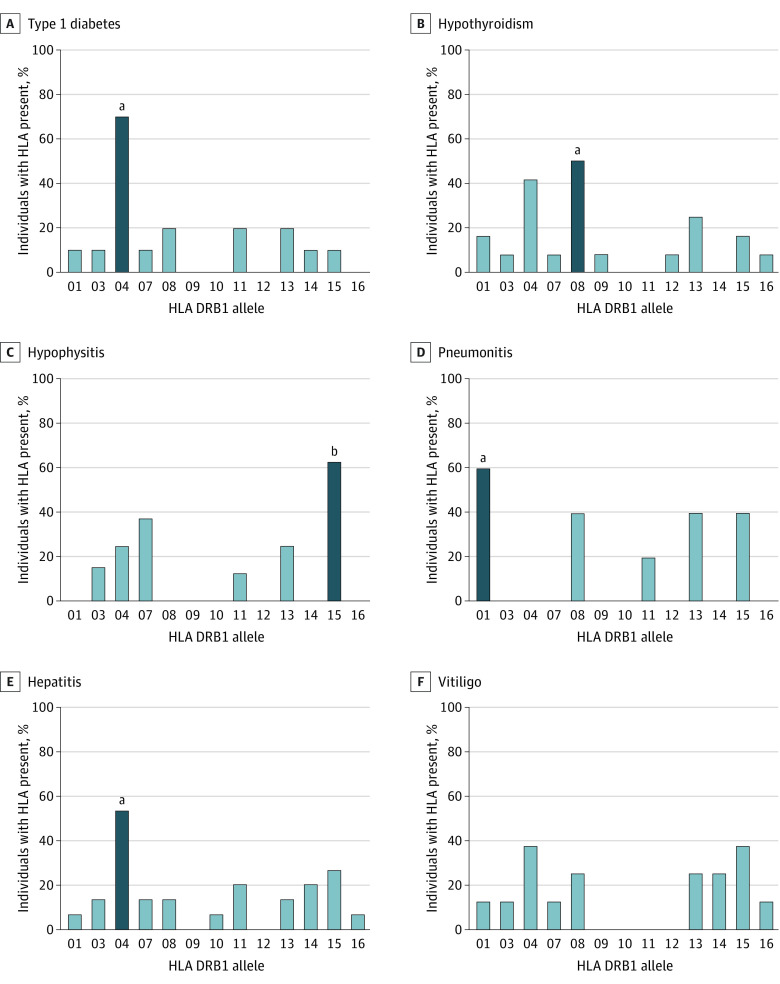
Association of Specific Human Leukocyte Antigen (HLA) Class II Genes With Immune-Related Adverse Events (irAEs) Immune-related adverse events included type 1 diabetes (in 10 patients), hypothyroidism (in 12 patients), hypophysitis (in 8 patients), pneumonitis (in 5 patients), hepatitis (in 15 patients), and vitiligo (in 8 patients). ^a^*P* < .01 using a Fisher exact test to compare patients with an irAE and a specific HLA-DR allele with those patients with no irAE and the same HLA-DR allele. ^b^*P* < .05 using a Fisher exact test to compare patients with an irAE and a specific HLA-DR allele with those patients with no irAE and the same HLA-DR allele.

Because specific HLA class II genes, especially DR alleles, are known to confer significant risk of autoimmune diseases outside the setting of cancer,^11,12,13,14,15^ our cohort underwent HLA-DR genotyping to investigate the possible genetic basis of irAE development. Specific HLA-DR alleles were associated with occurrence of certain irAEs when compared with patients who had any irAE with the same DR allele (Figure 2). We found 7 of 10 patients (70%) with type 1 diabetes had DR4; 6 of 12 (50%) with hypothyroidism had DR8; 5 of 8 (63%) with hypophysitis had DR15; 3 of 5 (60%) with pneumonitis had DR1; and 8 of 15 (53%) with hepatitis had DR4 (Figure 2A-E). Vitiligo was not associated with any specific HLA-DR allele but developed in the presence of many different alleles (Figure 2F). Additionally, several HLA associations remained when compared with the group without any irAE but having the same HLA-DR allele, including DR4 for type 1 diabetes (15 of 50 [30%] vs 70%; *P* = .01), hypophysitis with DR15 (13 of 50 [26%] vs 63%; *P* = .03), and DR8 with hypothyroidism (4 of 50 [8%] vs 50%; *P* = .003).

## Discussion

The immune checkpoints CTLA-4 and the PD-1/PD-L1 axis have an important physiologic role in normal adaptive immunity to render lymphocytes unresponsive after activation. In melanoma and other cancers, these checkpoints may be inappropriately suppressed, and blocking these checkpoints allows an immune response directed against cancer cells.^2^ In a subset of individuals, blocking negative immune regulation also leads to an immune response targeting normal self-tissues and thus development of an irAE.^3,5^ In this case-control study of patients with advanced melanoma, we demonstrate that individuals developing specific irAEs have an improved treatment response and prolonged survival compared with those without an irAE. This has been shown in other studies for both melanoma and non–small cell lung cancer.^16,17,18^

There is a need to understand the underlying pathophysiology of irAE development and identify easily measured markers to stratify patient risk. Herein, we used HLA antigen genotyping because these genes are known to confer risk for several diseases, including autoimmune disorders.^8^ We identified distinct HLA-DR alleles that were associated with a given irAE. Smaller studies have focused on a specific irAE showing associations of DR4 with type 1 diabetes,^19,20,21^ DR15 with hypophysitis,^22^ and DR11 with pneumonitis.^23^ We confirmed several of these associations in our cohort of patients, but the strength of our study is the finding of specific HLA-DR alleles conferring risk for 5 tissue-specific irAEs (eg, type 1 diabetes, hypothyroidism, hypophysitis, hepatitis, and pneumonitis). Because HLA class II molecules function to present processed peptides to CD4 T lymphocytes, our notable findings indicate that class II molecules may direct the immune system to target a specific self-tissue by presenting peptides from pancreatic islets, thyroid, pituitary gland, liver, or lungs when negative immune regulation is blocked.

Although we focused on HLA class II alleles, class I alleles have also been associated with response rates and survival after ICI therapy. The HLA-B alleles within the B44 supertype are associated with an increased treatment response and survival, whereas those with B*15:01 had poor outcomes.^24^ In a separate study,^25^ HLA-A*03 alleles were correlated with decreased survival. Taken together, these studies^24,25^ and our work presented herein indicate future studies are warranted to investigate complete HLA class I and II genotypes for cancer response rates, survival, and irAE development with ICI treatment.

Our study has both clinical and research implications. Common irAEs may increase morbidity, cause interruptions in ICI therapy, and in some cases lead to mortality, such as type 1 diabetes presenting with diabetic ketoacidosis. Human leukocyte antigen genotyping is robust, reproducible, and readily available in clinical laboratories. Assessing HLA alleles before ICI therapy may play a role in preventing common irAEs and better design future clinical trials.

### Limitations

Our study has some limitations. First, only HLA-DR alleles were typed, and other HLA class I and II alleles may have associations with specific irAEs. Second, the number of treatments and overall dose of ICI therapy were not collected and compared between participants with and without irAEs. Third, several irAEs were from small sample sizes. Future studies with a larger number of patients, including those with different cancer types, are necessary to confirm our findings.

## Conclusions

The findings of this case-control study suggest that developing certain irAEs after ICI therapy in advanced melanoma was associated with increased treatment response rates and improved survival compared with no development of adverse events. Distinct HLA-DR alleles are associated with given irAEs, thereby linking genetic predisposition to the targeted self-tissue with blockade of negative immune regulation.

## Notes

Supplement.
Data Sharing Statement
