# Supplementary material for: Analysis of Human Leukocyte Antigen DR Alleles, Immune-Related Adverse Events, and Survival Associated With Immune Checkpoint Inhibitor Use Among Patients With Advanced Malignant Melanoma
Source: JAMA Netw Open. 2022 Dec 13;5(12):e2246400. doi: 10.1001/jamanetworkopen.2022.46400 (PMC9856415; doi:10.1001/jamanetworkopen.2022.46400)
Supplement: Supplement. — Data Sharing Statement [file jamanetwopen-e2246400-s001.pdf]

Data Sharing Statement

Akturk. Analysis of HLA-DR Alleles, Immune-Related Adverse Events, and Survival Associated With Immune Checkpoint Inhibitor Use Among Patients With Advanced Malignant Melanoma. *JAMA Netw Open*. Published December 13, 2022. doi:10.1001/jamanetworkopen.2022.46400

Data
Data available: Yes
Data types: Deidentified participant data
How to access data: aaron.michels@cuanschutz.edu
When available: With publication

Supporting Documents
Document types: None

Additional Information
Who can access the data: Data will be made available to anyone requesting the data.
Types of analyses: Data will be made available for any purpose.
Mechanisms of data availability: Data will be made available to anyone requesting the data.
Any additional restrictions: No additional restrictions.
